# Patent Urachus and Associated Comorbidities in 101 Newborn Foals: A Retrospective Study

**DOI:** 10.1002/vms3.70379

**Published:** 2025-04-28

**Authors:** André Bernick, Judith Krohn, Axel Wehrend

**Affiliations:** ^1^ Veterinary Clinic for Reproductive Medicine and Neonatology Faculty of Veterinary Medicine Justus‐Liebig‐University Giessen Giessen Germany

**Keywords:** foal disorders, umbilical, umbilical cord, umbilical disease, urinary system

## Abstract

**Background:**

Patent urachus is a common disease in newborn foals. However, despite its frequency, studies investigating symptoms, comorbidities, treatment and prognosis in a large number of affected foals have rarely been conducted.

**Objective:**

This study aimed to describe the clinical symptoms, laboratory diagnostic findings and prognosis of foals with patent urachus.

**Method:**

Data from 101 foals with a patent urachus from 2006 to 2017 were analysed.

**Results:**

Patent urachus occurred at a frequency of 7.8% of all foals up to the 14th day of life during the study period. More colts (74.3%) than fillies (25.7%) were affected. In those with secondary urachal fistulae, patent urachus (91.1%) was significantly more diagnosed than persistent urachus (8.9%). Typical symptoms were a moist external umbilical environment (100%) and urine dripping from the external umbilical cord remnant during urination (75%). The average age at the time of diagnosis was 5.5 ± 3.2 days (median: 5 days, range: <1–13 days). Umbilical resection was performed in 29.7% of foals, whereas 70.3% were treated conservatively. In total, 67.3% of affected foals were discharged from the clinic. Foals that underwent umbilical resection were discharged in 76.7%, and those that underwent conservative treatment in 63.4% of cases. No typical laboratory diagnostic findings related to patent urachus could be determined. However, an elevated lactate concentration on the day of admission was linked to significantly worsened prognosis (*p* = 0.021). In 18.5% of the foals, which received surgery, a wound‐healing complication occurred at the abdominal suture. Further, 67% of the foals had one or more comorbidities. The presence of musculoskeletal disorders significantly worsened the survival prognosis (*p* = 0.037). In total, 46 foals were monitored for at least 6 months (6 months to 10 years) after discharge from the clinic. At this point, 93.9% of the foals were alive, and none developed any further health complications with the umbilicus or abdominal sutures.

**Conclusion:**

The primary result of this article is that patent urachus has a limited statistical influence on clinical parameters, laboratory values and prognosis. Comorbidities and/or sequelae worsen survival prognosis. The prognosis in the first 6 months after discharge was very good.

## Graphical abstract

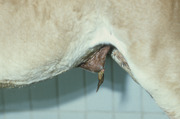

## 1 Introduction

Umbilical diseases are major causes of illness in newborn foals, among which patent urachus is common in neonatal foals (Graßl et al. 2017). On its own, patent urachus is rarely life‐threatening. However, in most cases, it is diagnosed with other comorbidities (Jung et al. 2008), from which life‐threatening conditions can develop. Patent urachus that does not respond to conservative therapy should be corrected surgically, as there is a risk of developing umbilical and bladder infections, which could be the starting point for secondary infections (e.g., joints) (Jung et al. 2008).

This study aimed to present data on the frequency, age, symptoms, comorbidities, progress and laboratory diagnostic findings of a larger case group of 101 affected foals at a clinic across a period of 12 years. Statistical calculations were performed for areas in which sufficient data were collected. In addition, foal health data were collected after discharge from the clinic. Statistical analysis of data regarding survival in clinics for foals suffering from patent urachus treated conservatively has not yet been covered in the literature.

## 2 Materials and Methods

### 2.1 Animals and Inclusion Criteria

All newborn foals admitted to or born in the clinic between 1 January 2006 and 31 December 2017, that were available for data collection, were included in the study.

All foals up to the age of 14 days with a confirmed diagnosis of patent urachus and urine leakage from the umbilicus were included. Diagnosis was confirmed by ultrasound examination or urachus probing.

### 2.2 Data Collection

Medical history and clinical findings were collected using standard study protocols, which remained unchanged throughout the study period.

The following data were collected: age at admission, age at diagnosis, breed, sex, treatment, hospitalization duration and foal outcomes. In addition, clinical symptoms, comorbidities, complications and, in the case of surgical treatment, wound‐healing complications were recorded.

The definitions for the three comorbidities, prematurity, hypogammaglobulinemia and Systemic Inflammatory Response Syndrome (SIRS), were as follows:

Prematurity: The inclusion criteria encompassed all foals born prematurely. By definition, such foals exhibited at least one of the following characteristics: a gestation period of less than 320 days, a short silky coat, soft ear cartilage (Koterba 1990) and/or unbroken premolars (Kablack et al. 2000).

Hypogammaglobulinemia was defined as a deficiency in class G immunoglobulins with a value of less than 800 mg/dL in the venous blood by at least 16 h postnatally (Sievert et al. 2022).

SIRS is a life‐threatening systemic inflammatory reaction. The clinical findings include disturbed general condition, fever, reduced ability to stand, petechial bleeding in the sclerae, injected episcleral vessels and reddened mucus membranes (Wong and Wilkins 2015). We defined SIRS criteria by the alteration of 2 or more of the following: a decreased or increased leukocyte count (<5.5 or >11.5 G/L), an increased heart rate (>100 beats per minute), an increased respiratory rate (>40 breaths per minute) and a decreased or increased temperature (<37.2°C or >38.6°C) (Roy et al. 2017).

For the evaluation of laboratory diagnostic findings in the venous blood, the following parameters were recorded: absolute erythrocyte count; haematocrit; absolute leukocyte count; pH value; concentrations of glucose and lactate; concentrations of ionized calcium, sodium, potassium and chloride; urea and creatinine concentrations and maternal immunoglobulin supply.

#### 2.2.1 Clinical Examination

General clinical examinations were performed directly following admission to the clinic or directly after birth, according to the segmental examination scheme for foals (Ennen and Wehrend 2010). Our physiological reference values for heart rate were 60–100 beats per minute, for respiratory rate 20–40 breaths per minute, and for rectal temperature 37.2–38.6°C (Hospes and Kolm 2011; Knottenbelt et al. 2007a). The appropriate external umbilical cord length after birth is 10–20 mm distal from the external umbilicus at the preformed rupture site (Jung 2011). Too short was defined as <10 mm and too long as >20 mm.

#### 2.2.2 Laboratory Diagnostics

Diagnostic tests were performed in the clinical's own laboratory. Diagnostic laboratory parameters were determined using venous blood samples. An EDTA tube (1.3 mL EDTA KE/1.3 tube from Sarstedt), a lithium heparin tube (1.3 mL lithium heparin LH tube from Sarstedt) and an arterial blood sampler (2 mL PICO 50 arterial blood sampler from Radiometer) were filled. Differential blood counts were determined using two different laboratory devices (2006–2013: CELL‐DYN 3500 DT from Abbott; as of 2014: ProCyte Dx from IDEXX). Both devices have been approved for use with horse blood. Venous blood from the EDTA blood sample tube (1.3 mL EDTA KE/1.3 tube from Sarstedt) was used for this purpose. To determine the concentrations of electrolytes (ionized calcium, sodium, potassium and chloride), pH value and lactate and glucose concentrations, the blood was analysed using an arterial blood sampler (2 mL PICO 50 arterial blood sampler from Radiometer) with two different devices (2006 to March 2009: ABL System 615 from Radiometer; as of April 2009: ABL 800 BASIC from Radiometer). Urea and creatinine concentrations were determined in EDTA (1.3 mL EDTA KE/1.3 tube from Sarstedt) or lithium heparin blood (1.3 mL lithium heparin LH tube from Sarstedt) using a blood chemistry system (Reflotron Urea Test and Reflotron Creatinine Test from Scil Animal Care Company). The IgG concentration was determined from lithium heparin blood (1.3 mL lithium heparin LH tube from Sarstedt) using a Snap IgG Test Kit (Snap Foal IgG Test Kit from IDEXX Laboratories).

#### 2.2.3 Treatment

Cases of patent urachus were treated conservatively or surgically. Conservative treatment was performed either with policresulen (Lotagen concentrate 360 mg/g from MSD Tiergesundheit, Germany) or using cryosurgery. Treatment was performed with the foal lying down and restrained. The umbilicus was disinfected in advance with 70% alcohol. During the treatment, 2 mL policresulen (Lotagen concentrate 360 mg/g from MSD Tiergesundheit, Germany) concentrate was instilled over a teat cannula on a syringe into the distal section of the urachus, as described by Jung et al. (2008). The treatment was repeated 2–12 times, on average 4–6 times daily, until the urachus was closed. Two medical devices (Askina Skin Freeze from B.Braun (Melsungen) or Cryoalfa Perfect from Cryoswiss (Basel)) were used for cryotherapy. The treatment was repeated at 1‐ to 2‐day intervals until the umbilicus dried. In some cases, local therapy with policresulen (Lotagen concentrate 360 mg/g from MSD Tiergesundheit, Germany) and cryotherapy have been used. In nine cases, the umbilicus was iodized. Surgical treatment was performed if conservative therapy was not successful after 5–7 days or if there were any indications for surgery, such as additional infected umbilical remnant, necrosis or abscess formation of the external umbilical remnant or the presence of an urachal lumen >6 mm. Umbilical resection with cystoplasty was performed under general anaesthesia.

To determine the foals’ outcome, owners whose foals were at least 6 months old were contacted by telephone, email or post to enquire about the foal's health.

#### 2.2.4 Data Analysis

Statistical analysis was performed in collaboration with the Unit for Biomathematics and Data Processing. The statistical program BMDP/Dynamic release 8.1 (Statistical Solutions Ltd., Ireland) was used. The primary outcome measure was survival at the clinic and after 6 months. The following factors were included as potential influencing factors in the model:

– Breed, sex and age;
– The presence of comorbidities (prematurity, hypogammaglobulinemia, SIRS, respiratory system diseases, gastrointestinal tract diseases, musculoskeletal system diseases and other umbilical diseases);
– Blood parameters: total leukocyte count, pH, lactate concentration and concentrations of sodium, potassium, chloride and ionized calcium.

Blood parameters on the day of admission were used for the statistical analysis of blood values. Only foals for which all parameters were available were used for the multiple logistic regression.

In the first step of the analysis, metric (age and blood parameters) and qualitative (breed, sex, comorbidities, treatment, wound‐healing complications, short‐term survival and long‐term survival) data were analysed. The arithmetic mean (*x̅*), the standard deviation (*s*), the median ($\tilde{x}$), the minimum (*x*_min_), the maximum (*x*_max_) and the range (*R*) were calculated for the metric values. For qualitative values, frequency counts were performed in tabular form. The second step involved identifying significant prognostic factors. The outcome variable was survival of the foal. This was performed separately with regard to fate in the clinic and survival at the age of 6 months. Stepwise logistic regression was used to examine raw correlations for each potential influence value. First, each potentially influencing variable was considered individually with respect to foal survival. All significant characteristics (*p* < 0.05) were analysed using a multiple logistic regression model. Only 86 foals with data on all parameters for analysis were included in the calculation of the multiple logistic regression.

## 3 Results

### 3.1 Incidence, Sex, Breed and Age

Patent urachus occurred in 101 of 1295 newborn foals (7.8%) up to 14 days of age, with a higher incidence in male foals than female foals (Table 1). The survival rate of the colts was higher (73.3%) than that for fillies (50%).

**TABLE 1:** Absolute and relative frequency of sex distribution (*n* = 101) for foals with patent urachus.

		Absolute	Relative in %
Sex	Male	75	74.3
	Female	26	25.7

Patent urachus was diagnosed in 66% warmblood foals and in 34% of other horse breeds. The breed was not documented in one case. The other breeds included 4 ponies, 6 thoroughbreds, 7 cold‐blood horses, 1 Friesian horse, 3 Icelandic horses, 3 Quarter Horses and 10 other breeds with 1 individual each.

**FIGURE 1:**
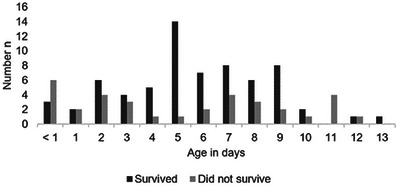
Age on the day of diagnosis for deceased and surviving foals with patent urachus (*n* = 101).

The age at the time of diagnosis was 5.5 ± 3.2 days (median 5 days, range: <1–13 days). Diagnosis was made by 7 days of age in 71.3% of foals (Figure 1). In nine foals (8.9%), persistent urachus was present on the day of birth; of these, 33.3% survived. Of the foals diagnosed with patent urachus between the 2nd and 10th days, 74.1% survived. Of the foals that were 11 or more days old, only 28.6% survived.

### 3.2 Clinical Symptoms

The findings of the general clinical examination are summarized in Tables 2 and 3. More the half of the foals (56.1%) were tachycardic, and 4.1% were bradycardic. Of the foals with tachycardia, physiological heart rate and bradycardia, 63.6%, 74.4% and 25.0% survived, respectively. More than the half of the foals (58.1%) had tachypnea, and 1.1% had a reduced respiratory rate. Of the foals with tachypnea and a physiological respiratory rate, 72.2% and 65.8% survived, respectively, whereas the one foal with a reduced respiratory rate did not survive. Over the half of the foals (52.1%) had a physiological rectal temperature, 25.5% had a fever, and 22.3% were hypothermic. Of foals with physiological body temperature, fever and hypothermia, 73.5%, 62.5% and 47.6% survived, respectively.

**TABLE 2:** Number of foals (*n*), arithmetic mean (*x̅*), standard deviation (*s*), median, range and reference value of initial heart rate, respiratory rate and rectal temperature of foals with patent urachus in the clinic.

	*n*	*x̅*	*s*	Median	Range	Reference value
Heart rate in beats (min)	98	108.9	29.8	109	28–200	60–100
Respiratory rate in breaths (min)	93	49.6	28.3	44	16–104	20–40
Rectal temperature in °C	94	37.8	1.5	38.1	32.4–40.0	37.2–38.6

**TABLE 3:** General conditions and ability to stand of foals with patent urachus at the time of admission to the clinic.

General condition	*n*	Good	Poor	Very poor	Comatose
	98	42	36	18	2
Ability to stand	*n*	Without help	With help	Lying down in upright position	Lying down in side position
	97	54	22	17	4

**FIGURE 2:**
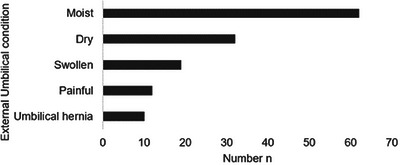
Condition of the umbilicus in foals (*n* = 94) with patent urachus on the day of admission or birth.

The external umbilical cord remnant length was documented in 70 foals at the time of diagnosis. In 77.1% of cases, the external umbilical cord remnant was physiologically torn; in 12.9% of cases, it was torn too short, and in 10% too long. On the day of admission or birth, the external umbilical cord remnant was moist in 66% of foals and dry in the rest. In 20.2% of cases, the external umbilical cord remnant was also swollen, and in 12.8%, it was painful. Further, 10.6% of foals presented with an umbilical hernia on admission. The condition of the external umbilical cord remnant on the day of admission or birth was not documented in seven foals (Figure 2).

**FIGURE 3:**
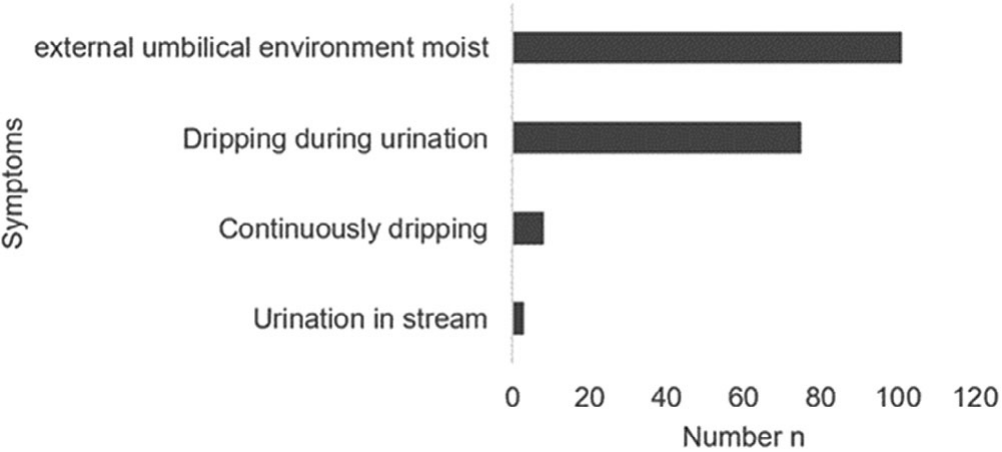
Urination from the umbilicus in foals (*n* = 101) with patent urachus.

A moist external umbilical environment was found in all foals at the time of diagnosis of the patent urachus. In 74.3% of foals, urine was observed to be dripping from the external umbilical cord remnant during urination. In 7.9%, the dripping was constant and independent of urination. In 3% of foals, there was a stream of urine from the opening of the external umbilical cord remnant during urination (Figure 3).

### 3.3 Comorbidities

In total, 95 of the 101 foals (94.1%) had at least 1 comorbidity, whereas 68 (67.3%) had 2 or more. The clinical incidence and survival rates of comorbidities in foals with patents in the clinic are summarized in Table 4. The statistical correlations for survival are listed in Table 9 (Section 3.9).

**TABLE 4:** Incidences, survival rates and statistical correlations for survival in the clinic of comorbidities in foals with patent urachus (*n* = 100).

Comorbidity	Incidence (%)	Survival rate with (%)	Survival rate without (%)
Prematurity	22	63.3	67.9
Hypogammaglobulinemia	41	92.7	49.2
SIRS	26	42.3	75.7
Respiratory diseases	32	50.0	75.0
Gastrointestinal tract diseases	42	76.2	60.3
Musculoskeletal diseases	36	50.0	76.6
Additional disease(s) of the umbilical cord	41	78.0	59.3

Abbreviation: SIRS, Systemic Inflammatory Response Syndrome.

The most common respiratory disease in foals with patent urachus that died or were euthanized was bronchopneumonia (93.8%), whereas the most common gastrointestinal diseases were diarrhoea, meconium impaction and pathologies of the small intestine. The 10 foals with patent urachus that did not survive suffered from diarrhoea (80%), meconium impaction (30%) and/or small intestine pathologies (30%). The most common musculoskeletal disease in foals with patent urachus was polyarthritis (50%). Of the foals with polyarthritis, 61.1% died or were euthanized.

Among the foals that died or were euthanized in the clinic, most suffered from more than one comorbidity (93.9%). Only two foals with patent urachus that did not survive suffered from one comorbidity: one from neonatal isoerythrolysis and the other from prematurity.

Of the foals with additional umbilical disease(s), 15 also had omphalitis simplex, 12 had umbilical hernias, 5 had omphalourachitis, 5 had omphaloarteritis, 2 had omphalophlebitis, 2 had omphaloarteritis and ‐phlebitis, and 1 had omphalourachitis and arteritis. Umbilical haematoma was diagnosed in four foals. No other umbilical cord disease was documented in one foal.

### 3.4 Laboratory Findings

The laboratory findings of the foals with patent urachus are summarized in Table 5. The erythrocyte count was within the reference range in 60.4% of foals, low in 18.8% and elevated in 20.8%. Of the foals with a low, within reference range and elevated erythrocyte count, 72.2%, 67.2% and 55.0% survived, respectively. The haematocrit was within the reference range in 57.3% of foals, low in 33.3% and elevated in 9.4%. Of foals with a low, within reference range and elevated haematocrit, 68.8%, 63.6% and 66.7% survived, respectively. Leukocytosis was present in 24% of the foals and leukopenia in 21.9%. Of foals with leukocytosis, leukopenia and with a total leukocyte count in the reference range, 56.5%, 57.1% and 73.1% survived, respectively. The pH value was within the reference range for 52.9% of foals, low for 40% and elevated for 7.1%. Of foals with a pH value that was too low, reference range and elevated, 47.1%, 75.5% and 50% survived. The lactate concentration was within the reference range in 18.6% of foals, low in 1.2% and elevated in 80.2%. Of foals with a lactate concentration within the reference range and elevated, 68.8% and 62.3% survived. One foal with low lactate concentration did not survive. The survival rate for foals with a lactate concentration between 1.7 and 5.0 mmol/L was higher (70.8%) than for foals with a lactate concentration over 5.0 mmol/L (42.9%). The sodium concentration was within the reference range in 92.9% of foals, low in 4.7% and elevated in 2.4%, among whom 50.0%, 63.3% and 50% survived, respectively. The potassium concentration was within the reference range for 79.1% of foals, low in 7% and elevated in 14%. Of foals with a low potassium concentration, in the reference range and with an elevated potassium concentration, 50%, 69.1% and 33.3% survived. The chloride concentration was within the reference range for 34.8% of foals, low for 47.8% and elevated for 17.4%, with these groups having survival rates of 27.3%, 75% and 33.3%, respectively. The ionized calcium concentration was within the reference range in 90.8% of foals, low in 2.3% and elevated in 6.9%. Both foals with a low ionized calcium concentration, 63.3% in the reference range and 50% with an elevated sodium concentration survived. The glucose concentration was within the reference range in 67.9% of foals, low in 30.9% and elevated in 1.2%. Of foals with a decreased, in the reference range and with an elevated glucose concentration, 48%, 67.3% and 100% survived. The creatinine concentration was within the reference range in 57.1% of foals, low in 16.7% and elevated in 26.2%. Of foals with a low creatinine, within reference range and with an elevated creatinine concentration, 42.9%, 66.7% and 45.5% survived, respectively. The urea concentration was within the reference range for 55.8% of foals and elevated in 44.2%. Of foals with a urea concentration within the reference range 66.7% and with an elevated urea concentration, 57.9% survived.

**TABLE 5:** Laboratory findings of foals with patent urachus in the clinic; number of foals (*n*), arithmetic mean (*x̅*), standard deviation (*s*), median, range and reference value.

	*n*	*x̅*	*s*	Median	Range	Reference value
Erythrocytes in T/L	96	9.6	2.0	9.8	3.5–14.3	8.0–11.0
Haematocrit in %	96	36.8	8.0	36.2	15.0–58.6	34–46
Leucocytes in g/L	96	9.2	5.8	8.3	0.6–34.1	5.5–11.5
pH value	85	7.34	0.1	7.37	7.0–7.63	7.36–7.43
Lactate in mmol/L	86	3.9	3.4	2.5	0.6–18.3	0.8–1.6
Sodium in mmol/L	85	135.8	7.4	135	117–162	125–150
Potassium in mmol/L	86	3.9	0.9	3.9	2.1–7.6	2.8–4.5
Chloride in mmol/L	23	96	9.7	94	78–117	95–105
Calcium in mmol/L	87	1.5	0.2	1.5	1.1–2.8	1.25–1.75
Glucose in mmol/L	81	6.8	3.4	7.6	0.3–21.6	6.0–12.5
Creatinine in µmol/L	42	149	127.6	109	44.2–662	71–159
Urea in mmol/L	43	8.4	7.5	5.6	3.3–35.4	3.3–6.7

### 3.5 Therapy

Umbilical resection was performed in 29.7% of foals (Table 6). The remaining foals received conservative treatment only at the umbilicus. Almost half of the foals (46.3%) with an additional umbilical disease underwent surgery, and only 18.3% of the foals which had no additional umbilical disease than patent urachus received surgery. Of the foals that were treated successfully conservatively, 85% were treated within 7 days and 57.5% within the first 3 days of hospitalization. Five (11.1%) of the foals treated conservatively were discharged with a patent urachus still present.

**TABLE 6:** Treatment of patent urachus in foals differentiated according to survival (surviving and deceased [absolute and relative]).

	Survived		
Treatment	Yes *n* (%)	No *n* (%)	Total *n* (%)
Conservative	45 (63.4)	26 (36.6)	71 (70.3)
Conservative/surgical	17 (81.0)	4 (19.0)	21 (20.8)
Surgical	6 (66.7)	3 (33.3)	9 (8.9)
Total	68 (67.3)	33 (32.7)	101 (100)

Survival rates of foals that were treated conservatively are shown in Table 7.

**TABLE 7:** Methods of conservative treatment for foals differentiated according to survival (surviving and deceased [absolute and relative]).

	Survived		
Therapy	Yes *n* (%)	No *n* (%)	Total *n* (%)
Policresulen	34 (66.7)	17 (33.3)	51 (71.8)
Cryotherapy	5 (41.7)	7 (58.3)	12 (16.9)
Policresulen + cryotherapy	3 (100)	0 (0)	3 (4.2)
Iod	3 (60)	2 (40)	5 (7.1)
Total	45 (63.4)	26 (36.6)	71 (100)

### 3.6 Complications

Five of 27 foals (18.5%) that underwent an umbilical resection developed wound‐healing complications. Two foals were euthanized during surgery. There were no data on wound healing for one foal that received surgery upon. One foal had a suture dehiscence of approximately 1 cm in length 13 days after surgery. One foal developed a medium‐sized seroma around the suture 2 days after the surgery. In one foal, the suture was swollen, with non‐purulent secretions, 12 days after the surgery. One foal developed a purulent wound‐healing complication of the abdominal sutures. One foal with ruptured urachus and urinary phlegmon had suture dehiscence. Overall, 67 foals developed one or more sequelae after diagnosis.

### 3.7 Hospitalization

The average length of hospitalization was 12.1 ± 7.9 days (median: 11, range: 1–32 days). Foals that were only in the clinic for 1 day died on the day of admission or were euthanized. The length of hospitalization was not documented in one foal. The average hospitalization for foals discharged in good health condition was 14.7 ± 7.5 days (median: 13, range: 3–32 days).

### 3.8 Progression After Discharge

At hospital discharge, 68 foals (67.3%) survived, among whom 60 (59.4%) showed no acute symptoms of a patent urachus or another comorbidity anymore, and 8 (7.9%) still showed acute symptoms of a patent urachus and/or another comorbidity. For four foals, the umbilicus was moist on the day of discharge. In one foal, the umbilicus was still moist, urine dripped from the umbilicus, and one foal had a low‐grade umbilical infection. In the two foals that underwent surgery, the sutures still had wound secretions. Seven foals (6.9%) died in the clinic, and 26 foals (25.7%) were euthanized due to a poor prognosis. No foals were euthanized because of a patent urachus. Euthanasia was performed in foals because of other comorbidities. Overall, 92.3% of these foals suffered from more than one comorbidity. The comorbidities of the euthanized foals are listed in Table 8.

**TABLE 8:** Age, general condition, ability to stand and comorbidities at the time of euthanasia in foals with patent urachus in the clinic.

Age in days	General condition	Ability to stand	Comorbidities	Cause of euthanasia
4	Comatose	Lying down in side position	Prematurity, hypogammaglobulinemia, SIRS, meconium impaction, polyarthritis, pneumonia and meningitis	Polyarthritis, meningitis
9	Poor	Lying down in side position	Meconium impaction, hypogammaglobulinemia, neonatal encephalopathy, entropion and thrombophlebitis left vena jugularis	Poor prognosis
19	Very poor	Lying down in side position	SIRS, hypogammaglobulinemia, pneumonia, polyarthritis and diarrhoea	Polyarthritis, very poor condition
5	Poor	Without help	SIRS, hypogammaglobulinemia, icterus, pneumonia and uroperitoneum	Uroperitoneum, euthanasia in surgery
9	Poor	No information	Hypogammaglobulinemia, SIRS, pneumonia and polyarthritis	Polyarthritis and poor condition
4	Poor	Without help	Meconium impaction, pneumonia and uroperitoneum	Uroperitoneum without permission of the owner for surgery
15	Good	Without help	Severe lameness in the left front leg with swollen carpus, elbow and shoulder and pneumonia	Severe lameness in the left front leg
13	Poor	With help	Severe dyspnoea, polyarthritis with severe tarsitis left, diarrhoea and pneumonia	Polyarthritis with severe tarsitis left
7	Poor	Without help	Uroperitoneum, coprostasis and pneumonia	In surgery because of atonic colon, severe impaction and pathological changes of the small intestine
16	Very poor	Lying down in upright position	Hypogammaglobulinemia, SIRS, hernia scrotalis, hernia inguinalis, pneumonia, icterus and diarrhoea	Hernia inguinalis after first surgery with bowel prolapse and remaining SIRS
19	Poor	Lying down in side position	SIRS, flexor tendon shortening of the front legs on both sides, severe lameness right front leg and pneumonia	Musculoskeletal diseases
10	Poor	With help	SIRS, hypogammaglobulinemia, neonatal encephalopathy, seizures, entropion, polyarthritis, carpus valgus, conjunctivitis, pneumonia and diarrhoea	Poor condition, seizures, polyarthritis
7	Good	With help	Stringhalt und carpus varus in the front legs, hyperextension in the hind limbs and tarsitis	Musculoskeletal diseases
4	Comatose	Lying down in side position	Hypogammaglobulinemia, SIRS, neonatal encephalopathy, dyspnoea, overreaching in the hind limbs and colic with dilated intestinal loops	Comatose and very poor prognosis
15	Poor	No information	Diarrhoea, overreaching in the front legs, pneumonia and uroperitoneum	Uroperitoneum (defect in the right ureter), euthanasia in the second surgery
13	Poor	Lying down in side position	Hypogammaglobulinemia, flexor tendon shortening of the front legs on both sides, pneumonia, right front leg of the horse with severe laminitis and loss of the hoof capsule	Right front leg of the horse with severe laminitis and loss of the hoof capsule
23	Poor	Lying down in side position	Polyarthritis all four limbs severe overreaching and pneumonia	Musculoskeletal diseases
22	Very poor	Lying down in side position	Prematurity, hypogammaglobulinemia, SIRS, diarrhoea and polyarthritis	Very poor condition and polyarthritis
7	Poor	Lying down in side position	SIRS, pneumonia with dyspnoea, pleural effusion, pleurisy, shortening of the front legs on both sides	Pleural effusion, pleurisy
10	Very poor	Lying down in side position	Prematurity, hypogammaglobulinemia, SIRS, polyarthritis and pleural effusion	Polyarthritis and pleural effusion
19	Very poor	With help	Prematurity, hypogammaglobulinemia, flexor tendon shortening in the front legs, severe overreaching in the hind limbs, diarrhoea, trush, neurological failures and pericardial effusion	Neurological failures, pericardial effusion
28	Very poor	No information	Hypogammaglobulinemia, SIRS, polyarthritis, systolic cardiac murmur, abscess on the thoracic wall and diarrhoea because of cryptosporidiosis and colic	Severe colic without permission of the owner for surgery (ultrasound finding: highly dilated intestinal loops)
3	Poor	Without help	Rupture of the urachus and phlegmon by urine of the abdominal skin	Tripping of urine out of the wound suture after surgery
9	Very poor	Lying down in side position	Neonatal isoerythrolysis	Neonatal isoerythrolysis
4	Very poor	No information	Prematurity, neonatal encephalopathy, hyperextension all four limbs, partial atelectasis of the lungs, corneal opacity, trush and colic	Severe colic without permission of the owner for surgery
10	Very poor	Lying down in side position	Prematurity, anaemia and leukopenia	Very poor condition

Abbreviation: SIRS, Systemic Inflammatory Response Syndrome.

Of the 68 discharged foals, 49 were traced and subsequently monitored. There were 46 foals (93.9%) that reached an age of at least 6 months. Three foals (6.1%) were euthanized before this age. One foal was euthanized at the age of 3 months due to incurable ataxia, one was euthanized at the age of 5.5 weeks due to severe colic of unknown origin, as it did not have surgical authorization, and one was euthanized at the age of 24 days because of a generally poor condition and cramps due to diarrhoea caused by Cryptosporidium infection.

Thirty‐nine foals were alive at the age of at least 1 year. Four foals were sold at the age of 6 months, and one was sold at the age of 7 months, meaning that no further information was available on these animals. One foal was euthanized at 6.5 months due to severe colic, as it did not have surgical authorization. One foal was euthanized at the age of 1 year because of a pelvic fracture. Five horses were aged 1 year at the time of the study, six horses were 2 years, seven horses were 3 years, five horses were 4 years, two horses were 5 years, six horses were 6 years, two horses were 7 years, three horses were 8 years, two horses were 9 years, and one horse was alive at the age of 10 years.

None of the 49 foals presented complications at the umbilicus or surgical site after discharge. The five foals that were discharged with a patent urachus were successfully treated conservatively in their home stables, as were the foals that were discharged with a low‐grade umbilical infection. The two foals that still had wound secretions from the sutures were successfully treated conservatively for a few days.

### 3.9 Statistical Correlations for Survival

In multiple logistic regression analysis, simultaneous musculoskeletal disease and elevated lactate levels were found to be significantly correlated with non‐survival (*p* < 0.05) in the clinical setting (Table 9). The presence of SIRS and elevated lactate levels showed a significant correlation with non‐survival 6 months after discharge (*p* < 0.05). Notably, patent urachus was not correlated with survival in the multiple regression analysis.

**TABLE 9:** *p* values for parameters of foals with patent urachus (*n* = 86) in statistical correlations for survival in the clinic and 6 months after discharge.

Parameter	*p* value survival in the clinic	*p* value survival 6 months after discharge
Sex	0.141	0.171
Breed	0.066	0.306
Age	0.558	0.397
Prematurity	0.224	0.703
Hypogammaglobulinemia	0.42	0.776
Sirs	0.058	0.027
Respiratory diseases	0.654	0.547
Gastrointestinal tract diseases	0.3	0.146
Musculoskeletal diseases	0.037	0.104
Additional disease(s) of the umbilical cord	0.128	0.285
Leucocyte concentration	0.409	0.128
Venous pH value	0.245	0.495
Lactate concentration	0.021	0.045
Sodium concentration	0.676	0.916
Potassium concentration	0.868	0.695
Concentration of ionized calcium	0.91	0.983
Therapy	0.276	0.983
Wound‐healing complications	0.52	0.755

Abbreviation: SIRS, Systemic Inflammatory Response Syndrome.

## 4 Discussion

Patent urachus is a common disease affecting newborn foals. However, this topic has rarely been comprehensively discussed in the literature. Indeed, there have been only five case studies, with a limited number of cases of 8–40 foals (Adams and Fessler 1987; Jung et al. 2008; Reig Codina et al. 2019). In one case study, only surgically treated foals were included (Reig Codina et al. 2019).

The pathogenesis and treatment of patent urachus have been well described (Bernick et al. 2021), with many general statements regarding the symptoms. Data on the frequency and severity of symptoms and healing process have only been reported in one study (Jung et al. 2008). The short‐term prognosis is stated in existing case studies. However, there is a lack of information in the literature regarding breed distribution, comorbidities, laboratory parameters and long‐term prognosis.

Data collection in this study was retrospective. The disadvantages of a retrospective study include incomplete and partially subjective basic data, which means that there is a risk of insufficient prerequisites for the application of statistical methods. This risk was addressed using the same standard protocol throughout the study period. However, some information was still missing, as indicated in Section 3 at the relevant points. The scope of examinations and treatment procedures is influenced not only by medical necessity but also by the consent of the owner. Examples include the decision of whether a foal should receive surgery and the scope of laboratory diagnostics. As such, it should be noted that the present study has the disadvantages of retrospective data collection but nevertheless provides new findings compared to the previous literature because of the number of cases, statistical data processing and follow‐up enquiries on foals’ progress.

The frequency of foals presenting with patent urachus up to 14 days of age was 7.8% during the study period; this exceeds the figure of 4.5% reported in another case study (Graßl et al. 2017). However, in that study, only foals up to the age of 10 days were included. It should be noted that patent urachus, a secondary urachal fistula, was significantly more frequently diagnosed (91.1%) than persistent urachus at birth (8.9%). In one case study, patent urachus was more commonly observed at birth (27.5%) (Reig Codina et al. 2019). However, this study only considered foals with umbilical resections, whereas no foals were treated conservatively. The frequency of occurrence of a secondary urachal fistula indicates the need to check newborn foals for the presence of an urachal fistula in the first few days of life.

Physiologically, the urachus in foals closes during or immediately after birth (Hackett et al. 1982; Knottenbelt et al. 2007b). The following causes may be attributed to the failure of the closure of the urachus: genetic defects, tearing of the umbilical cord above the predilection site, traumas (e.g., through the mare during trying to stand up) and increased intravesical or intraabdominal pressure caused by tenesmus (e.g., in association with meconium impaction) (Jung 2011). Persistent urachus may be caused by torsion‐related partial obstruction of the umbilical cord in utero, with dilatation of the urachus. This results in delayed closure (Adams and Fessler 1987; Richardson 1985; Turner et al. 1982). Additionally, an above‐average external umbilical cord remnant length can cause patent urachus (Knottenbelt et al. 2007b; Schott 2012). Cutting the umbilical cord instead of allowing it to naturally tear can also hinder urachus closure (Knottenbelt et al. 2007b). A persistent urachus occurs more commonly in premature foals because the umbilical cord structures are not yet fully developed; thus, independent closure of the blood vessels and urachus is hardly possible (Jung 2011). Patent urachus occurs more often in foals with sepsis or in immunocompromised and weak foals. A correlation between the increased and prolonged lying phases is suspected (Knottenbelt et al. 2007b; Jung 2011). Patent urachus may result from infections of the umbilical vein, arteries and/or urachus, such that the urachus is reopened by inflammatory processes (Adams and Fessler 1987).

Patent urachus was more common in male than in female foals in the present study, although the difference did not reach statistical significance. This result agrees with those of other case studies, in which patent urachus occurred more frequently in male foals, at a ratio of 63%–85% (Adams and Fessler 1987; Bäumer 1997; Jung et al. 2008). One possible explanation for this could be that colts have narrower pelvises, which can lead to increased pressure on the bladder during the birth process and delay the closure of the urachus around birth. A patent urachus can also develop because of increased pressure caused by tenesmus in the foal, for example, in the case of meconium obstruction. It also occurs more frequently in male than in female foals (Knottenbelt et al. 2007b).

The affected foals showed a typical distribution of horse breeds in clinics in Germany. The average age of the foals at the time of diagnosis with patent urachus was 5.5 ± 3.2 days. Almost two‐thirds of foals were admitted to the clinic for other underlying diseases and developed a patent urachus later. This result is consistent with previous age data of 0–16 days (Adams and Fessler 1987; Jung et al. 2008). Patent urachus occurs more frequently than persistent urachus at birth and usually develops only when the dried umbilicus falls off, around the 5th–7th day. Umbilical inflammation facilitates this process (Adams and Fessler 1987).

Of the foals with a patent urachus, just over half were found to have tachycardia and tachypnea on the initial examination. Additionally, slightly more than half of the patients had physiological rectal temperature. In most cases, it should be considered whether heart rate, respiratory rate and temperature are also influenced by the environment, other underlying diseases and comorbidities, and whether they are age‐dependent in the neonatal foal (Knottenbelt et al. 2007b). As such, no general statement can be made regarding the three vital parameters related to patent urachus. The same applies to the general condition and ability to stand of foals with a patent urachus. Up‐to‐date comparative values from the existing literature are not known at the time of writing.

For more than three‐quarters of the affected animals, the external umbilical cord remnant was torn at a normal physiological location at the time of diagnosis. The literature states that an above‐average external umbilical cord remnant length can cause a patent urachus (Knottenbelt et al. 2007b; Schott 2012). However, this risk factor was not confirmed in the present study. The primary symptom at the time of diagnosis was moistness of the umbilicus due to urine drainage, and in most cases, urine dripped from the umbilicus during urination. This is consistent with the data in the literature (Bostedt 2017; DeNotta 2022; Knottenbelt et al. 2007b). Only in few cases was persistent dripping of urine independent of urination or urination in a stream from the umbilicus observed. Thus, it can be deduced that navel moisture was the most important symptom. As this symptom is not particularly obvious compared to urination from the umbilicus, close observation of the foal is required to detect the disease at an early stage.

A high percentage (94.1%) of foals with patent urachus had one or more comorbidities. Only 6 of 101 foals over a period of 11 years showed no comorbidities in the clinic. A prior case study involving 40 foals showed similar results (Reig Codina et al. 2019). In this study, 20 foals had patent urachus without umbilical infection, of which 15 had other comorbidities. The other 20 foals had patent urachus and umbilical infections. Thirteen patients had additional comorbidities. Only 5 of the 40 foals with patent urachus in the clinic over a 12 years period had an uncomplicated patent urachus. There may be other results for foals with patent urachus treated in their home stables. This aspect should be examined in future investigations. In addition, local umbilical infection has been described as a risk factor for patent urachus (Adams and Fessler 1987; DeNotta 2022), and infections of the urachus and/or umbilical structures, septicaemia and metastatic joint diseases can worsen the prognosis (Litzke and Siebert 1990).

No laboratory diagnostic parameters are typically associated with a patent urachus. The values are typically influenced by comorbidities, rather than the presence of a patent urachus. However, it is advisable to perform a differential blood count for all foals with a patent urachus and to determine the status of maternal immunoglobulins to detect and treat any signs of comorbidities early, such as infections or hypogammaglobulinemia. It has previously been described that hypogammaglobulinemia in conjunction with umbilical resection due to patent urachus and/or umbilical infection increases the risk of postoperative complications (Reig Codina et al. 2019). This correlation was not demonstrated in the present study, presumably because all foals with hypogammaglobulinemia had compensated this deficit after plasma transfusions.

More foals that underwent umbilical resection survived than those treated conservatively only. This may be due to the fact that an open and possibly infected umbilicus caused by patent urachus can serve as an entry point for secondary infections that worsen prognosis. However, it should be mentioned that this difference in survival did not reach significance. Very sick foals may not undergo surgery because of their altered physiology and will have a poorer prognosis. A case study of 40 foals with patent urachus also reported good prognosis after umbilical resection (Reig Codina et al. 2019). However, conservative treatment is justified. Of the foals that were treated conservatively and discharged in good health, 85% were cured within 1 week and more than half within 3 days. The likelihood of a successful conservative therapy decreases after a treatment period over 7 days. Only 33.3% of foals that were treated conservatively for longer than 7 days were successfully cured.

Many authors recommend the prophylactic use of broad‐spectrum antibiotics for the systemic therapy of patent urachus (Adams and Fessler 1987; Hackett et al. 1982; Jung 2011; Knottenbelt et al. 2007b; Schott 2012) to reduce the risk of umbilical infections, cystitis and septicaemia. According to one author, a patent urachus that occurs in the first hours of life should only be treated if it persists until the second day of life (Freytag 1976). According to Adams (1990), in many cases, the urachus closes on its own, provided that there are no additional comorbidities and the external umbilical remnant is kept clean. These findings have been discussed in other studies. On the basis of this, the urachus can close spontaneously, but in most cases, treatment is indicated to ensure rapid and complete closure (Hackett et al. 1982) or immediate therapeutic intervention is recommended, as complications such as umbilical infections, sepsis and secondary joint diseases can significantly worsen the prognosis (Litzke and Siebert 1990). For infection prophylaxis, the external umbilical cord remnant can be repeatedly immersed in a 0.5% chlorhexidine solution. In addition, it should be kept clean, and the surrounding skin areas should be covered with Vaseline to protect them from irritation caused by urine (Knottenbelt et al. 2007b). During treatment, particular attention should be paid to maintaining the stable dry and clean (Neil 2011). Conservative treatment attempts can be made, as long as no inflammatory changes in the urachus or umbilical cord remnants are present (Adams and Fessler 1987) and the lumen of the urachus is at least 6 mm (Jung et al. 2008). Cryosurgery, sclerotherapy and cauterization have been described to close the opening of the urachus (Jung et al. 2008). Sclerotherapy for treatment of a patent urachus is no longer recommended in the literature (Jung 2011; Knottenbelt et al. 2007b), as it can lead to rupture of the urachus, uroperitoneum (Ford and Lokai 1982) and/or a severe umbilical and urachal inflammatory reaction (Jung 2011). However, these complications are rarely observed in practice (Knottenbelt et al. 2007b), as observed in this study.

In the present study, the diameter of the urachal lumen was not considered, as described previously (Jung et al. 2008). This could be taken as an indicator of prognosis and the decision on whether to treat a patent urachus conservatively or surgically. It is advisable to perform an umbilical resection if the urachal lumen is ≥6.0 mm, as the prognosis for the success of conservative treatment is poor (Jung et al. 2008).

The length of hospitalization of 14.7 ± 7.5 days for those foals discharged in good health was primarily dependent on comorbidities and sequelae. The foal with the shortest hospitalization of 3 days was treated conservatively and also suffered from meconium obstipation, which was also treated conservatively. The longest hospitalization was 32 days; this foal was born prematurely, presented with meconium obstipation and developed multiple sequelae, including moderate bronchopneumonia, diarrhoea and unilateral thrombophlebitis. These circumstances made it necessary for the foal to stay in the clinic for a longer period. Foals that underwent umbilical resection were hospitalized for at least 8 days or longer. No comparable data are available in the literature regarding to the length of hospitalization of foals with patent urachus following surgical treatment.

The outcome of foals with patent urachus depends primarily on other comorbidities and sequelae, such as polyarthritis, bronchopneumonia or diarrhoea. Foals with no diseases other than a patent urachus survived. Statistical correlations for survival were analysed using multiple logistic regression. Previous studies have not used this procedure. The advantage of multiple logistic regression is that both quantitative and qualitative variables (and combinations) could be analysed if the number of individuals per risk factor is small.

Simultaneous musculoskeletal disease and elevated lactate concentration have been shown to be significantly correlated with non‐survival (*p* < 0.05) of foals with patent urachus in the clinic. A case study of 82 foals that underwent umbilical resection (40 with patent urachus) showed that the presence of septic arthritis and/or physitis also showed a significant correlation with failure to survive (Reig Codina et al. 2019). In addition, it has been reported that infections of the urachus and/or umbilical structures, septicaemia and metastatic joint diseases can worsen prognosis (Jung 2011; Litzke and Siebert 1990). A case study of 643 foals from 13 clinics showed that the probability of death increased for every 1 mmol/L increase in lactate concentration on the day of admission (Borchers et al. 2012). This study refers to the total population of newborn foals presenting with various diseases and not specifically to foals with a patent urachus.

Only four foals showed colic symptoms after discharge from the clinic later in life, of which three underwent an umbilical resection. It is possible that colic was secondary to the first abdominal cavity surgery. As there are no exact findings regarding the causes of colic, no precise statement can be made regarding its connection with abdominal cavity surgery. Overall, it should be noted that the medium‐ and long‐term prognoses for foals with patent urachus are very good.

## 5 Conclusion

Patent urachus is a common umbilical disease in newborn foals. Early diagnosis and treatment are important to minimize sequelae, which can reduce the likelihood of survival. The main result of this study was that patent urachus had a limited statistical influence on clinical parameters, laboratory values and prognosis. Comorbidities and/or sequelae worsen the prognosis in the clinic. The prognosis for the first 6 months after discharge was very good.

## Author Contributions

Axel Wehrend proposed and designed the study. André Bernick collected and analysed data. Axel Wehrend and André Bernick drafted and edited the manuscript. Judith Krohn examined a large number of the foals. All authors read and approved the final manuscript.

## Ethics Statement

The authors confirm that the ethical policies of the journal, as noted on the journal's author guidelines page, have been adhered to.

## Conflicts of Interest

The authors declare no conflicts of interest.

### Peer Review

The peer review history for this article is available at https://publons.com/publon/10.1002/vms3.70379.

## Data Availability

The data that support the findings of this study are available from the corresponding authors upon reasonable request.
